# Surgical Management of Choledocholithiasis in a Patient After Bariatric Surgery at a Rural Hospital

**DOI:** 10.7759/cureus.110984

**Published:** 2026-06-16

**Authors:** Atley Moberly, Shane Moore, Shane Monnett

**Affiliations:** 1 General Surgery, Midwestern University Arizona College of Osteopathic Medicine, Glendale, USA; 2 General Surgery, Yavapai Regional Medical Center, Prescott, USA

**Keywords:** choledocolithiasis, common bile duct exploration, effect of bariatric surgery, gallbladder disease, rural hospital

## Abstract

Choledocholithiasis is a serious complication of gallstone disease, and its management can be challenging in patients with altered gastrointestinal anatomy following bariatric surgery. We report the case of a 73-year-old woman with a history of unspecified bariatric surgery who presented with abdominal pain and evidence of a 14-mm common bile duct stone and ductal dilation. Initial endoscopic retrograde cholangiopancreatography (ERCP) was unsuccessful due to altered anatomy and an inability to traverse the duodenojejunal limb. In a rural hospital without advanced endoscopic options, open common bile duct exploration (OCBDE) was performed, successfully removing the stone. The patient recovered without complications. This case highlights the limitations of ERCP in post-bariatric surgery patients and the continued importance of OCBDE as a definitive, potentially lifesaving intervention in resource-limited or rural environments.

## Introduction

Gallstone disease is estimated to affect 10-15% of the adult population in developed countries, with a predicted rise in prevalence due to the ongoing obesity epidemic [1]. While many patients with gallstones may remain asymptomatic, some can develop complications related to stone migration. One such complication is choledocholithiasis, defined as the presence of gallstones in the common bile duct (CBD), the structure responsible for transporting bile from the gallbladder into the duodenum. Most cases result from migration of gallstones from the gallbladder, rather than de novo formation in the duct [2]. The clinical significance of untreated choledocholithiasis lies in its potential to cause serious complications, including acute cholangitis, gallstone pancreatitis, and obstructive jaundice [3]. In one retrospective observational study, among 123,990 discharges with choledocholithiasis, 41% had complicated disease, including 12% with acute cholangitis and 31% with acute pancreatitis [4]. Prompt recognition and management are therefore essential to prevent morbidity and mortality.

Patients who have undergone bariatric surgery represent a particularly high-risk group for gallstone formation and subsequent choledocholithiasis. Rapid weight loss postoperatively alters cholesterol metabolism and disrupts enterohepatic circulation, promoting cholesterol gallstone formation [5]. In one systematic review, the risk of de novo gallstone formation after bariatric surgery was 20.7% [6]. Because of this increased risk, the American Association of Clinical Endocrinologists/American College of Endocrinology, The Obesity Society, American Society for Metabolic and Bariatric Surgery, Obesity Medicine Association, and American Society of Anesthesiologists developed the 2019 clinical practice guidelines, which recommend postoperative ursodeoxycholic acid as prophylaxis for gallstone development [7]. Management of choledocholithiasis in this population can additionally present unique challenges due to surgically altered anatomy. Endoscopic retrograde cholangiopancreatography (ERCP) is a standard therapeutic approach for extraction of CBD stones and can be performed either before or after cholecystectomy [8]. However, bariatric procedures, including Roux-en-Y gastric bypass and duodenal switch, can make standard ERCP difficult due to altered gastric anatomy and limited access to the duodenum. In these cases, alternatives to ERCP include laparoscopic CBD or open CBD exploration, robotic-assisted laparoscopic CBD exploration, laparoscopic-assisted ERCP, or endoscopic ultrasound-directed transgastric ERCP (EDGE) [9]. Selection of the optimal intervention depends on available expertise, patient factors, and institutional resources.

In addition to the considerations made for this specific patient population, the management of choledocholithiasis can be further complicated by a rural practice setting. Rural hospitals may have limited access to necessary supplies and advanced endoscopic interventions such as ERCP. In such cases, surgical CBD exploration remains a critical option for definitive treatment. In this context, we present a case of successful retrieval of gallstones via open CBD exploration in a bariatric surgery patient in a rural setting. This case highlights the role of operative CBD exploration as a safe and effective treatment when endoscopic access is not possible, and the importance of surgical adaptability when endoscopists or advanced endoscopic resources are not available.

## Case presentation

A 73-year-old woman presented with a 2-day history of postprandial abdominal discomfort that began after eating a large, fatty meal. She reported experiencing similar pain in the past after overeating. The patient attempted to induce emesis, with little relief of her discomfort. Past medical history was significant for obesity complicated by hypothyroidism, hypertension, hyperlipidemia, and prediabetes. Her obesity had previously been managed with an unspecified bariatric procedure, which was not a Roux-en-Y gastric bypass or sleeve gastrectomy. The patient's gastric surgery had been performed many years prior, and she was unable to provide further details regarding the procedure. Additional past surgical history included splenectomy at the time of bariatric surgery.

On arrival to the ED, her vital signs were as follows: temperature 36.5°C, heart rate 66 beats per minute, respiratory rate 16 breaths per minute, and blood pressure 119/64 mmHg. Her weight was 88 kg. Physical examination revealed a soft, non-distended abdomen with mild right upper quadrant tenderness and a negative Murphy’s sign. No scleral icterus was noted.

**Figure 1:**
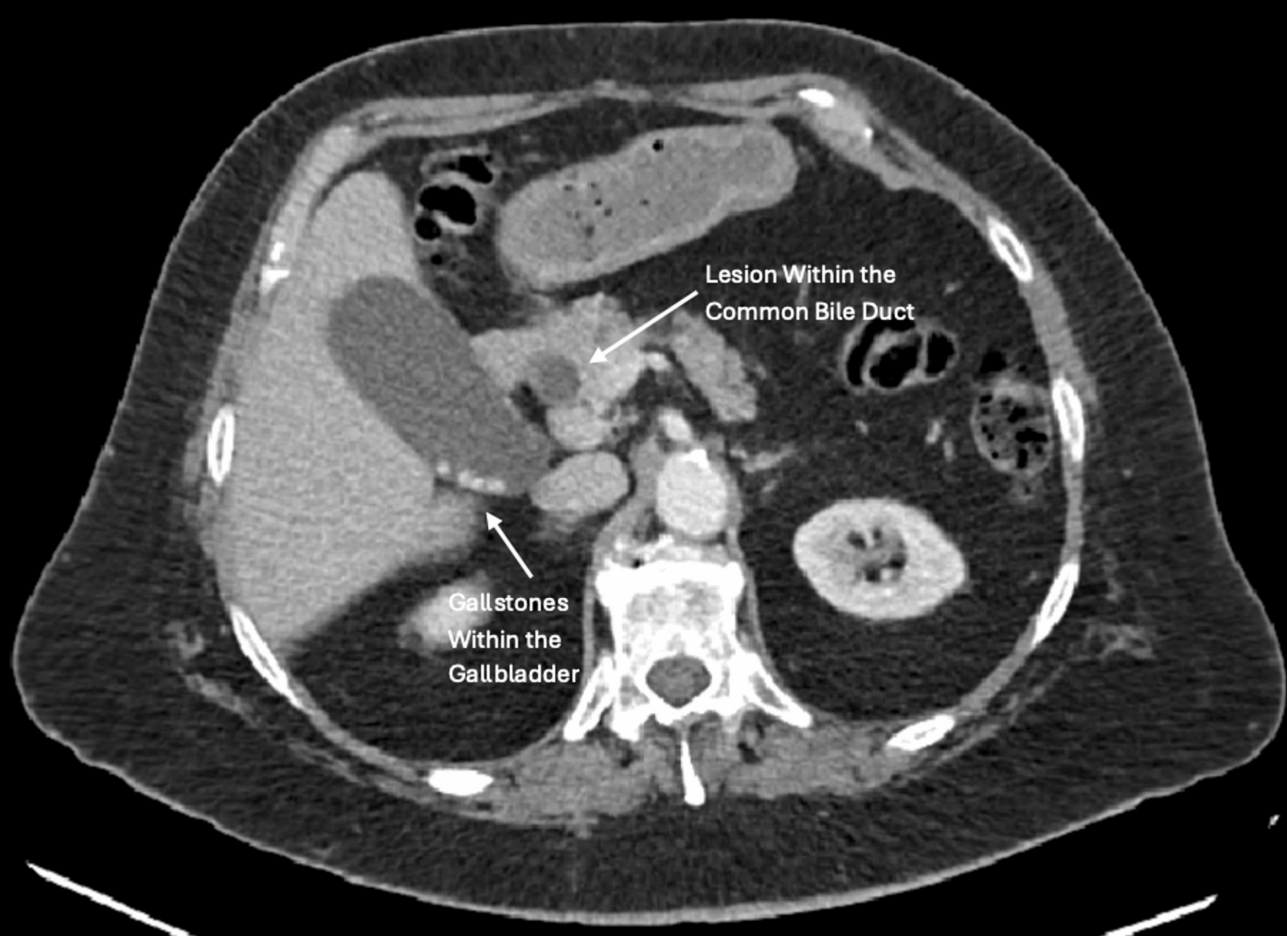
Computed tomography of the abdomen and pelvis. Gallstones are noted within the gallbladder, with a lesion identified within the common bile duct.

CT of the abdomen and pelvis performed at the referring facility demonstrated a 14-mm lesion in the CBD, dilation of the CBD to 13 mm, and moderate intrahepatic biliary dilation. The gallbladder was distended with gallstones (Figure 1). Laboratory studies on admission (Table 1) revealed a hemoglobin level of 12.4 g/dL (reference range: 12.0-16.0), hematocrit of 37.0% (reference range: 37.0-47.0%), and WBC count of 19,200 cells/µL (reference range: 4,200-10,000). Liver function testing showed total bilirubin of 2.9 mg/dL (reference range: 0.2-1.1), alanine aminotransferase (ALT) of 336 U/L (reference range: 10-49), aspartate aminotransferase (AST) of 421 U/L (reference range: 15-37), and alkaline phosphatase of 140 U/L (reference range: 46-116).

**Table 1:** Laboratory values on admission.

Test	Value	Reference range
Hemoglobin	12.4 g/dL	12.0-16.0 g/dL
Hematocrit	37.00%	37.0-47.0%
Leukocytes	19,200 cells/µL	4,200-10,000 cells/µL
Total bilirubin	2.9 mg/dL	0.2-1.1 mg/dL
Alanine aminotransferase (ALT)	336 U/L	10-49 U/L
Aspartate aminotransferase (AST)	421 U/L	15-37 U/L
Alkaline phosphatase	140 U/L	46-116 U/L

**Figure 2:**
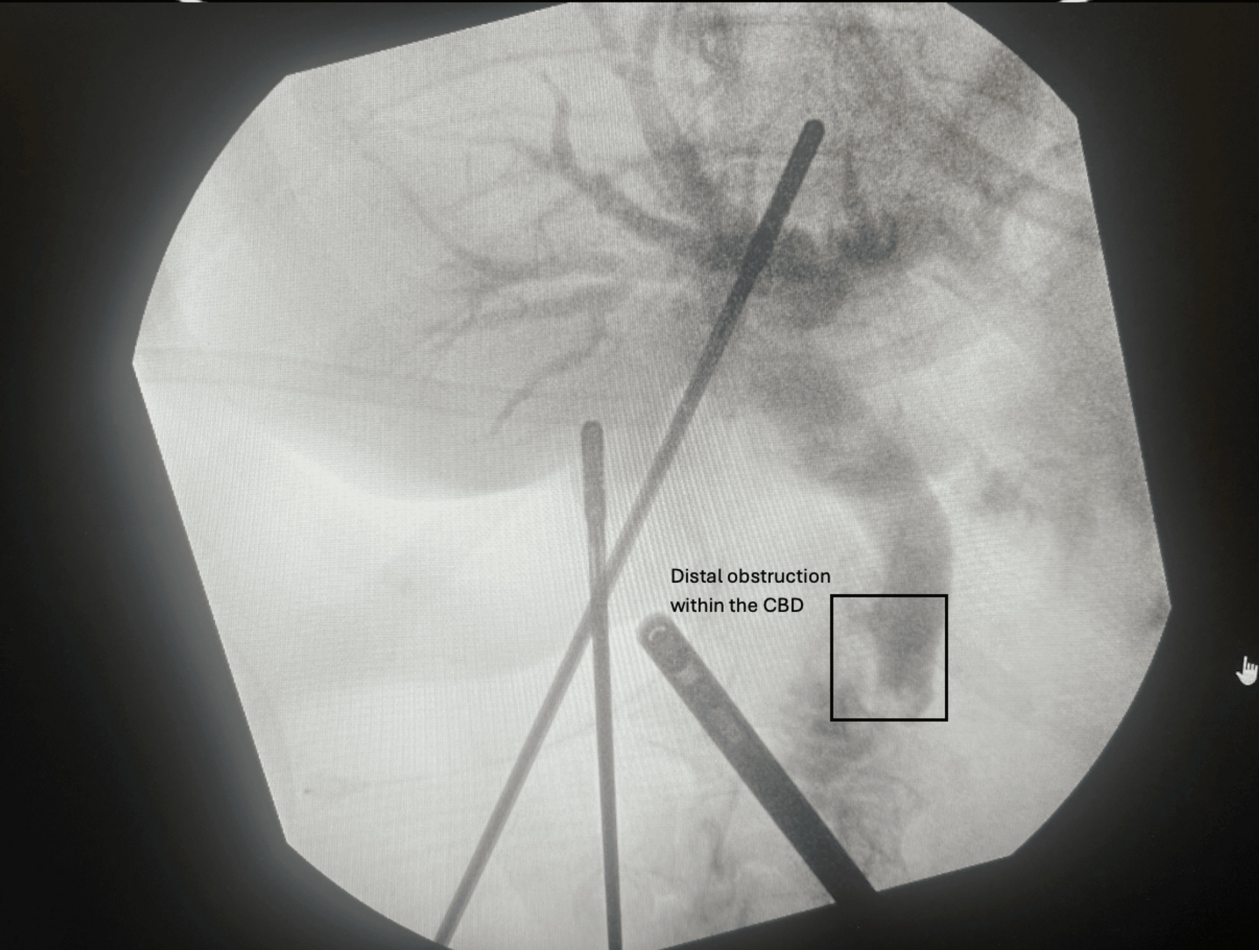
Intraoperative cholangiogram. The intraoperative cholangiogram demonstrates contrast-filled, dilated intrahepatic and extrahepatic biliary ducts. There is abrupt cutoff of contrast at the distal common bile duct, with a visible filling defect. No contrast is seen entering the duodenum, confirming distal obstruction. CBD: Common bile duct.

The patient underwent robotic-assisted laparoscopic cholecystectomy with intraoperative cholangiography, which revealed common bile duct (CBD) obstruction with multiple stones located posterior to the duodenum (Figure 2). Intraoperatively, extensive scar tissue and adhesions were noted overlying the stomach, limiting further dissection and preventing definitive characterization of the patient's bariatric surgery. Given the possibility of recanalization across the staple line permitting endoscopic access to the excluded stomach and duodenum, ERCP was attempted the following day for stone retrieval. Endoscopy demonstrated a small gastric pouch with limited access to the gastric antrum and duodenum, findings consistent with prior bariatric surgery. As a result, ERCP could not be completed, and no specimens were obtained. Given the persistent choledocholithiasis, the patient subsequently underwent open CBD exploration. Multiple CBD stones were successfully identified and removed (Video 1). Primary repair of the CBD was performed, and intraoperative cholangiography confirmed ductal patency. The patient tolerated the procedure well without immediate complications, and her postoperative recovery was unremarkable.

**Video 1:** Extraction of gallstones from the common bile duct.

## Discussion

Choledocholithiasis remains a significant complication of gallstone disease and, if untreated, can lead to serious outcomes, including obstructive jaundice, acute cholangitis, or gallstone pancreatitis [3]. ERCP is generally considered a first-line intervention for both diagnosing and removing stones from the CBD due to its minimally invasive nature and high success rates [8]. However, in patients with surgically altered anatomy, such as those who have undergone Roux-en-Y gastric bypass, standard ERCP frequently becomes futile [10]. Alterations in anatomy include sharp angulation and stenoses, which make passage through the anastomotic site difficult. In the case of Roux-en-Y gastric bypass, there is also the risk that the Roux limb may exceed the reach of the duodenoscope [11].

The American Society for Gastrointestinal Endoscopy (ASGE) recommends a risk-stratification algorithm categorizing patients as low, intermediate, or high risk for CBD stones based on clinical, laboratory, and imaging findings. High-risk features include stones visualized on imaging, total bilirubin greater than 4 mg/dL with a dilated CBD, or evidence of ascending cholangitis. These findings warrant preoperative ERCP. Patients with intermediate-risk features, including abnormal liver function tests, age greater than 55 years, or ductal dilation, are recommended to undergo further evaluation with endoscopic ultrasound (EUS), magnetic resonance cholangiopancreatography (MRCP), or cholangiography performed during cholecystectomy [12]. In our case, the intraoperative cholangiogram demonstrated obstruction within the CBD. When positive, laparoscopic CBD exploration (LCBDE) or postoperative ERCP should be considered. Notably, the guidelines do not provide specific recommendations for management following failed ERCP. Importantly, the guidelines emphasize that management should be tailored to local resources, stating that ERCP or LCBDE should be selected based on available surgical and endoscopic expertise [12]. This framework directly supports consideration of surgical CBD exploration in settings where advanced endoscopic options are limited.

Although choledocholithiasis was identified on the intraoperative cholangiogram, immediate LCBDE was deferred because the exact nature of the patient's prior bariatric procedure was unknown. The patient reported a history of "gastric stapling," raising the possibility of vertical banded gastroplasty. With this procedure, there was a possibility of recanalization through the staple line, which would still permit conventional endoscopic access to the duodenum. Given the lower invasiveness of ERCP and its shorter anticipated anesthesia time at our institution compared with LCBDE, ERCP was considered a reasonable initial strategy. Additionally, extensive scar tissue encountered during cholecystectomy increased the technical complexity and potential risk of injury associated with immediate laparoscopic bile duct exploration. Following unsuccessful ERCP, open CBD exploration was selected as the most reliable approach for definitive duct clearance.

Alternatives to standard ERCP include EDGE and laparoscopic-assisted ERCP. In a meta-analysis, the EDGE procedure was shown to have high clinical success in patients with gastric bypass, at a rate of approximately 95.9%. Laparoscopic-assisted ERCP was found to have a slightly lower clinical success rate of 92.9% [13]. However, these approaches require specialized equipment and skilled endoscopists, which may not be available in rural hospitals. In a U.S. nationwide study, 73% of hospitals without ERCP access were rural, and these hospitals had worse outcomes related to acute biliary pancreatitis, a complication of untreated choledocholithiasis [14].

When ERCP approaches are unsuccessful or inaccessible, surgical CBD exploration offers an alternative treatment. LCBDE is often preferred due to its minimally invasive nature; however, it can be technically challenging in patients with prior abdominal surgeries due to adhesions and altered anatomy [15]. De Raeymaeker X et al. demonstrated that LCBDE can still be successfully performed in patients with prior gastric surgery, though with increased technical complexity requiring advanced laparoscopic expertise [16]. Similarly, Wever R et al. highlighted that while LCBDE outcomes can be favorable, success is highly dependent on surgeon experience and institutional resources [17].

Importantly, limitations in laparoscopic expertise and equipment remain a significant barrier. A systematic review by Marks and Al Samaraee reported that approximately 5-8% of LCBDEs require conversion to open exploration [18]. This underscores that even in centers attempting minimally invasive approaches, open common bile duct exploration (OCBDE) remains a critical fallback when laparoscopic techniques fail or are not feasible. In cases where laparoscopic approaches are limited by adhesions, anatomical complexity, or resource constraints, OCBDE remains a reliable and effective option. Prior studies have demonstrated a ductal clearance rate of 90% with OCBDE versus a clearance rate of 84% in the LCBDE group [19]. In the present case, extensive perigastric adhesions and scar tissue prevented safe characterization of the patient's prior bariatric anatomy without undertaking a potentially high-risk dissection that could have resulted in enterotomy or necessitated revision of a previous bypass. Furthermore, multiple CBD stones were identified posterior to the duodenum. Compared with open exploration, laparoscopic or robotic extraction of multiple distal stones may be technically more demanding and require substantially longer operative times. Although minimally invasive CBD exploration can be effective in selected patients, open exploration was favored because it offered a more efficient and familiar means of achieving definitive ductal clearance in this complex clinical scenario. OCBDE therefore provided a reliable treatment option in a patient with previous abdominal surgeries, adhesions, altered anatomy, and failed ERCP.

This case highlights the continued relevance of OCBDE as a critical intervention. While minimally invasive techniques continue to evolve, open exploration remains an essential and dependable approach when modern endoscopic and laparoscopic options are either not feasible, unsuccessful, or unavailable.

## Conclusions

This case highlights the complex management of choledocholithiasis in a bariatric surgery patient presenting to a rural hospital. Altered gastrointestinal anatomy following bariatric procedures can preclude successful ERCP, a standard approach for common bile duct stone removal. After rapid weight loss and changes in bile composition following bariatric surgery, the risk of gallstone formation is significantly increased. In resource-limited rural settings where specialized endoscopic techniques such as EDGE or laparoscopic-assisted ERCP are not available, repeated attempts at endoscopy may carry additional risks without improving outcomes. In such contexts, OCBDE remains an essential, definitive intervention. In bariatric patients presenting to a rural hospital, it is crucial to maintain surgical proficiency in OCBDE to provide timely management of complex biliary disease.
